# High-Bandpass Filters in Electrocardiography: Source of Error in the Interpretation of the ST Segment

**DOI:** 10.5402/2012/706217

**Published:** 2012-06-21

**Authors:** F. Buendía-Fuentes, M. A. Arnau-Vives, A. Arnau-Vives, Y. Jiménez-Jiménez, J. Rueda-Soriano, E. Zorio-Grima, A. Osa-Sáez, L. V. Martínez-Dolz, L. Almenar-Bonet, M. A. Palencia-Pérez

**Affiliations:** ^1^La Fe Research Institute, Avenue Campanar 22, 46009 Valencia, Spain; ^2^Department of Cardiology, La Fe University Hospital, Bulevar Sur S/N, 46026 Valencia, Spain; ^3^Department of Electronic Engineering, Polytechnic University of Valencia, Camino Ver S/N, 46022 Valencia, Spain

## Abstract

*Introduction*. Artifactual variations in the ST segment may lead to confusion with acute coronary syndromes. *Objective*. To evaluate how the technical characteristics of the recording mode may distort the ST segment. *Material and Method*. We made a series of electrocardiograms using different filter configurations in 45 asymptomatic patients. A spectral analysis of the electrocardiograms was made by discrete Fourier transforms, and an accurate recomposition of the ECG signal was obtained from the addition of successive harmonics. Digital high-pass filters of 0.05 and 0.5 Hz were used, and the resulting shapes were compared with the originals. *Results*. In 42 patients (93%) clinically significant alterations in ST segment level were detected. These changes were only seen in “real time mode” with high-pass filter of 0.5 Hz. *Conclusions*. Interpretation of the ST segment in “real time mode” should only be carried out using high-pass filters of 0.05 Hz.

## 1. Introduction

 The existence of electrocardiographic changes in ST segment level is frequently due to acute coronary syndromes. Elevation of the ST segment above 0.1 mV on two adjacent leads, as well as the appearance of complete left bundle block (CLBB), in the presence of angina pain lasting more than 30 minutes within the first 12 hours of the development of symptoms is classical indications of urgent reperfusion therapy [1].

 Most biological signals must be processed for adequate recording [2]. Signal acquisition, signal processing, and high-pass filters may distort the shape of the recorded signal [3, 4] and sometimes may cause electrocardiographic changes simulating myocardial ischemia [5–9].

In this paper the frequently described, but not so well-known, distortion of the ST segment due to the technical recording characteristics of the electrocardiography system is profusely analyzed. The objective is to give a clear and graphic explanation of this aspect for a better understanding from a clinical point of view.

## 2. Material and Method

 We have analyzed 45 consecutive patients seen in the Cardiology Department for scheduled electrocardiograms (EGG); 19 patients presented for clinical cardiologic followup while the rest were referred to preoperative ECG for various noncardiac pathologies. All were asymptomatic, and none had a history of acute coronary syndrome in the previous six months.

 The low-frequency cutoff (high-pass filter) was set at 0.05 and 0.5 Hz. Low-pass filters were set at 40, 100, and 150 Hz (high-frequency cutoff). In this way, twelve ECG traces per patient were made, using “auto” and “manual” recording modes with the following filter configurations: 0.05–40 Hz, 0.5–40 Hz, 0.05–100 Hz, 0.5–100 Hz, 0.05–150 Hz, and 0.5–150 Hz. All records were collected with the same electrocardiograph (Hewlett Packard 1700 A). Auto mode means “computer processed” while manual mode expresses “real-time print (no delay),” in this equipment.

When ST segment changes were recorded in a given patient, the corresponding deviation was measured with respect to the rest of recordings obtained from the lead exhibiting the most accentuated changes.

**Figure 1:**
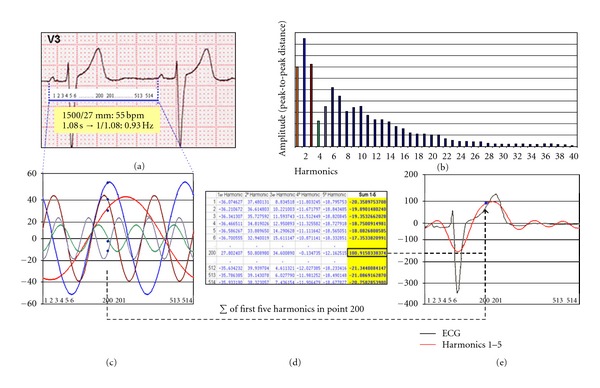
(a) The ECG corresponding to Figure 7, which was used to obtain a sample of the signal, recording the coordinates of 514 points in one PP cycle. The cardiac frequency is 55 bpm, which implies an RR cycle or PP cycle of 1.08 s. The first harmonic represented in (b) and (c) in red colour has a frequency of 0.9 Hz (1/1.08), and on the basis of this frequency the others are multiples (1.8, 2.7 Hz) of the first. (b) Spectrum of frequencies, by amplitude, corresponding to the 169 harmonics into which the signal was decomposed. The zone corresponding to the first 40 harmonics, which are those that contribute to the basic shape of the ECG signal, is shown—the harmonics beyond the first 25–30 having a very low amplitude. (c, d) Graphic and tabular presentation of the first 5 harmonics. Each one is composed of 514 coordinates corresponding to the PP cycle sample previously defined. Compare (b) and (c) (matching colors). The frequency spectrum is the amplitude representation (peak-to-peak distance) of each one of the harmonics mentioned. The sum of the 5 waves in each one of the 514 points is presented in tabular form in the last column of (d) and in graphic form superimposed in red colour on the digitized ECG signal in (e).

**Figure 2:**
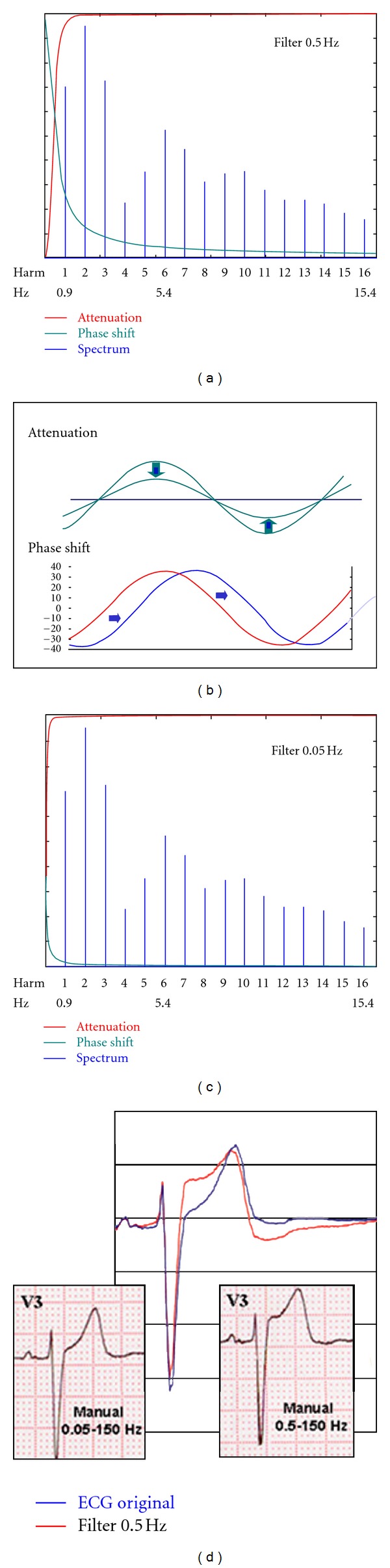
(a) The application of high-pass filters with cut-off frequencies of 0.5 Hz produces minimal attenuation of the amplitude of the first harmonic in the frequency spectrum of our signal but yields phase shifts that affect up to the fifth or sixth harmonic (corresponding to a frequency of ~5 Hz). (b) Attenuation of a wave is a decrease of the peak-to-peak amplitude. Phase shift is a displacement of the wave. (c) High-pass filters of 0.05 Hz do not cause attenuation or phase shifts in the frequency spectrum of the signal. (d) Here is shown the correspondence between the digital signals obtained by scanning a trace before and after application of a 0.5 Hz high-pass filter, using the MATLAB 5.0 program, with those obtained in real traces by manual recording with high-pass filters of 0.05 and 0.5 Hz. “Manual” mode means real-time print.

According to the Fourier transform theory, ECG signal, like any repetitive signal, can be considered a sum of a series of sine waves (components of different frequency and amplitude) named harmonics. In order to explain the ST segment alterations produced with the different filters and recording mode, a spectral analysis of the ECG was made. Based on MATLAB 5.0 software, a discrete Fourier transform analysis was carried out of a signal composed of 40 repetitions of a PP cycle, previously scanned and defined on the basis of 514 coordinate points obtained with the program Corel Draw 7.0, which implied a sampling frequency equivalent to 400 Hz. MATLAB 5.0 provided the complex coefficients corresponding to the harmonics composing the signal, of which the first 169 were selected. From each of the harmonics, defined by an amplitude and a phase, the corresponding coordinates were obtained for the 514 points previously defined for the PP cycle, verifying that point-by-point summation of the successive harmonics reconstituted the original signal (Figure 1). In order to analyze the effect of high-pass filtering on signal shape, first-order high-pass filters with cut-off frequencies of 0.05 and 0.5 Hz were applied to the constructed signal, using the MATLAB 5.0 program. Likewise, the complex coefficients were obtained for the first 169 harmonics that constituted the signal after the application of the corresponding filter, and the resulting shape obtained by the summation of these harmonics was compared with that of the original ECG recorded with the mentioned filters (Figure 2).

## 3. Results

**Figure 3:**
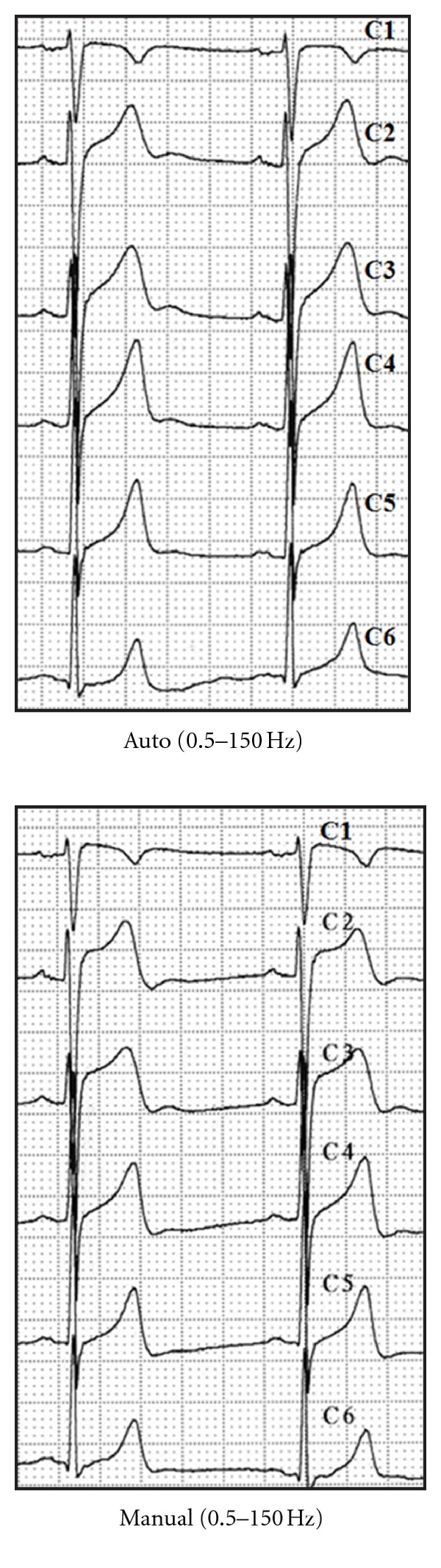
Auto versus manual ECG recording. Variations in the ST segment. ST elevation in V1–V3 on adopting manual mode ECG recording with 0.5–150 Hz bandpass filters. Auto means computer processed. Manual means real-time filtered.

**Figure 4:**
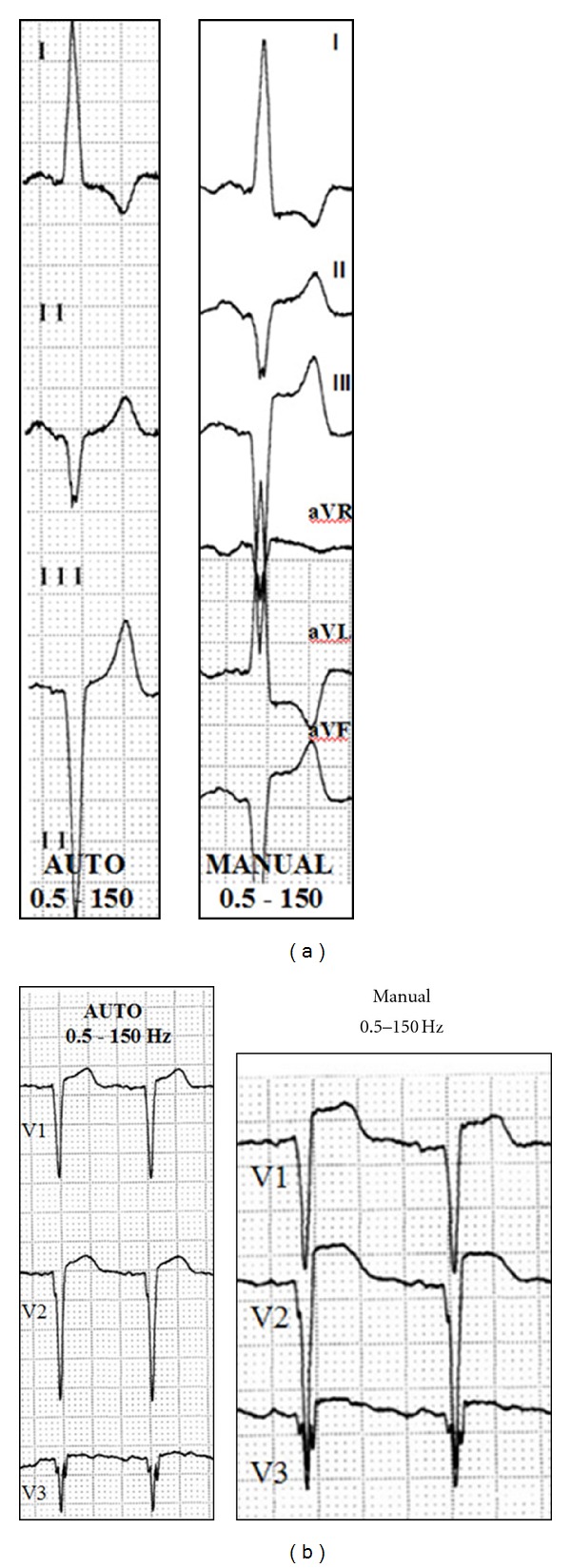
Anomalies in patients with structural heart disease. (a) ECG of a patient with marked left ventricular enlargement. Recording in manual mode yields ST segment depression in I and aVL and inferior aspect elevation that is particularly marked in lead III. (b) ECG of a patient with residual anteroseptal necrosis. An elevated ST segment appears in V1–V3 in the manual recording mode. Auto and manual mode (see Figure 3).

**Figure 5:**
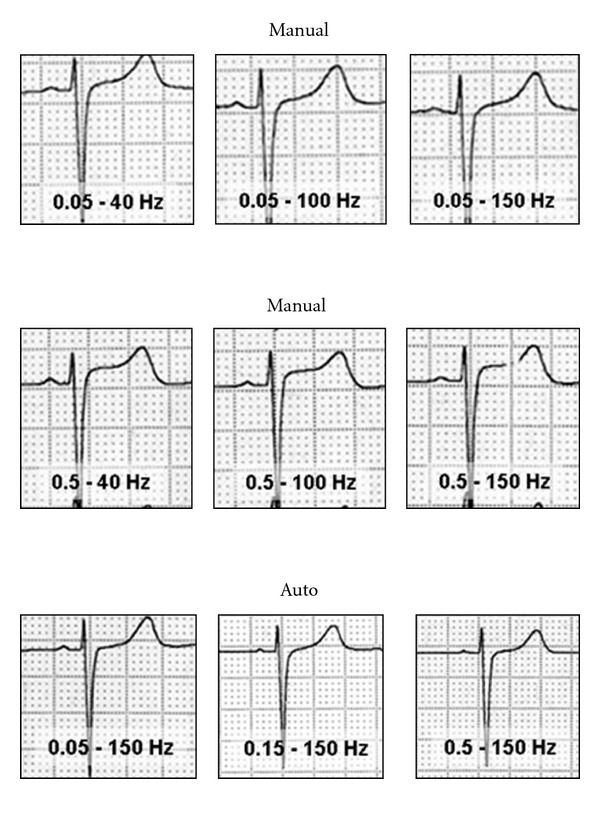
Variation of the ST segment according to the filter used. Lead V2. In the upper part no ST variations were observed in changing the low-pass filters (40, 100, or 150 Hz). The variations in the ST segment were only produced in the manual recording mode with high-pass filters of 0.5 Hz, regardless of the low-pass filter used (intermediate zone). In auto mode (lower part), no changes in the ST segment were produced. Auto and manual mode (see Figure 3).

**Figure 6:**
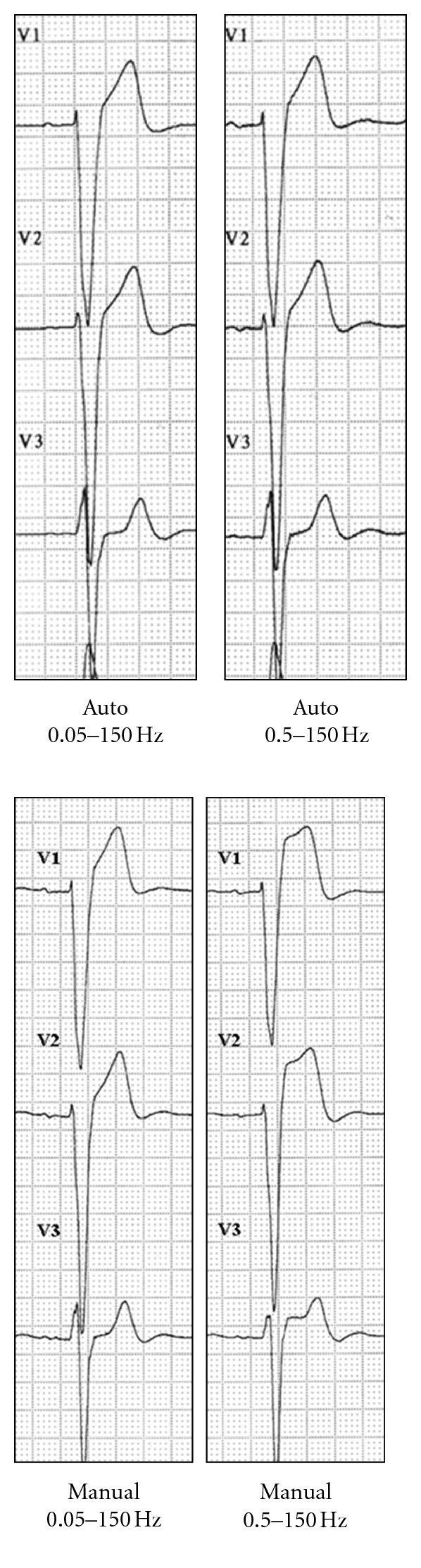
Alterations in the ST segment in the presence of left bundle block. In autorecording mode and regardless of the high-pass filter used, the shape of the ST segment was not affected. On the other hand, 0.5 Hz high-pass filters in manual mode caused a considerable elevation of the ST segment in V1–V3. Auto and manual mode (see Figure 3).

 In 42 patients (93%), abnormal ST segment elevations (generally corresponding to the leads with a predominant S wave, V1–V4, and/or inferior leads) or depressions (corresponding to the leads with a predominant R wave, I, aVL, V4–V6) were observed, which were considered clinically significant (Figures 3 and 4). These changes were only observed in the manual recording mode (real-time print) with high-pass filters of 0.5 Hz, regardless of the low-pass filter employed—40, 100, or 150 Hz—(Figure 5). The variation of the J point in traces exhibiting maximum deviation with respect to the baseline recordings ranged from 1.5 to 9 mm (average 3 mm). The most pronounced changes were seen in patients with a wide QRS and a slight basal deviation of the J point (left bundle block, pacemaker beats, Figure 6, patients with an anterior hemiblock or left ventricular enlargement, and also in patients with a normal ECG, normal J point, and a concave upper ST segment in V1–V3.

**Figure 8:**
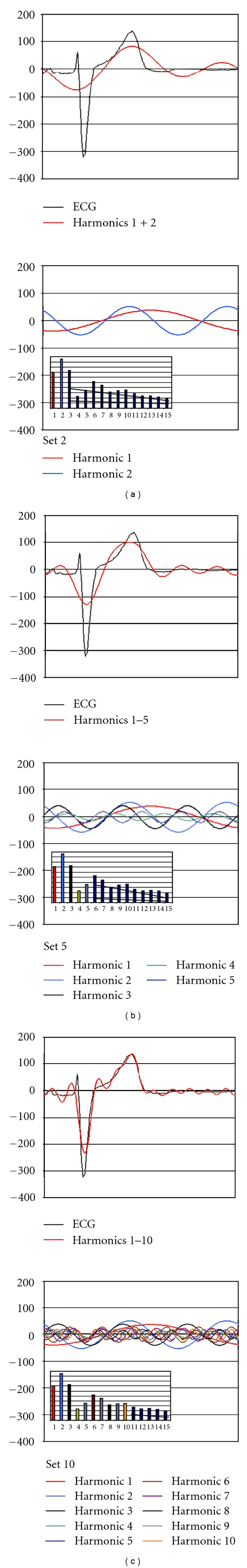
Reproduction of the ECG signal with summation of the successive harmonics. (a) In the lower part are shown the first two harmonics of the frequency spectrum in which the signal was decomposed. In the upper part, superimposed on the digitized ECG signal, the curve in red colour represents the point-by-point sum of the harmonics indicated. (b, c) Likewise, a representation of the sum of the first 5 and 10 harmonics, respectively.

**Figure 9:**
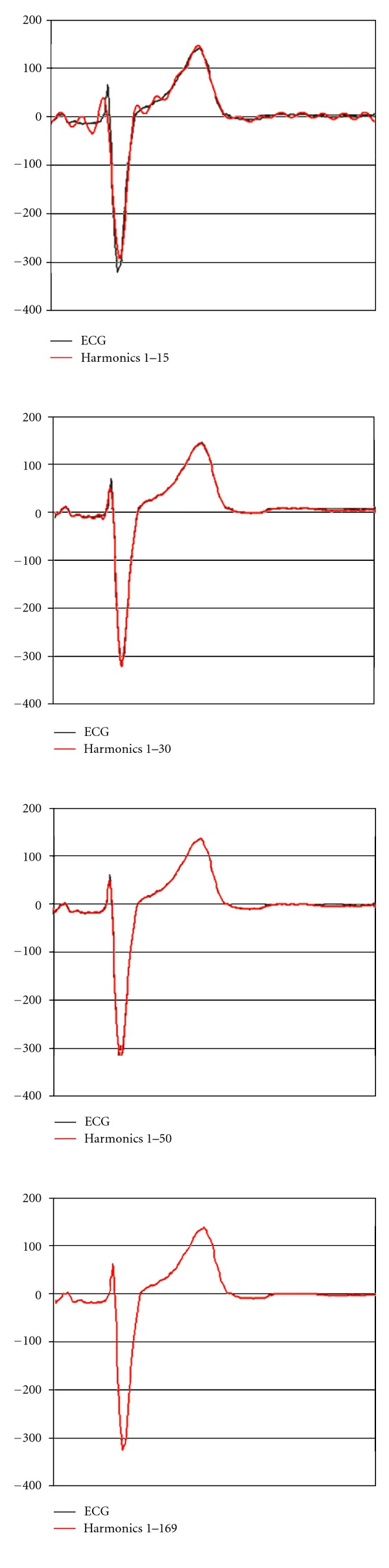
Reproduction of the ECG signal with the sum of the successive harmonics. The sum of the first 30–50 harmonics yielded a near-perfect reproduction of the ECG signal at the expense of small changes in QRS amplitude. The sum of the 169 selected harmonics reproduces in identical form the original signal.

 The reconstruction of the electrocardiogram from the sum of the successive harmonics into which the signal was decomposed (see Figure 1) is shown in Figures 8 and 9. The sum of the first 30 harmonics [0.9–27 Hz] reconstructs the ECG almost perfectly, except for small changes in QRS amplitude, though the shape of the ST segment and T wave is already identical to the original.

**Figure 10:**
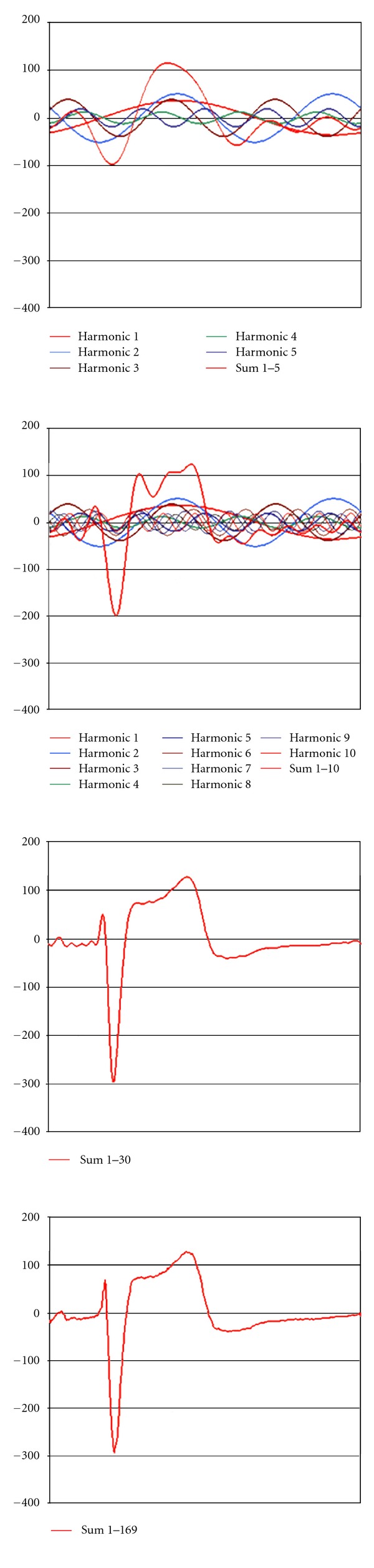
Application of a high-pass filter with a cut-off frequency of 0.5 Hz using the MATLAB 5.0 program applied to the digitized ECG signal. The recomposition of the ECG by means of the sum of the successive harmonics (5, 10, 30, and 169), obtained from the application of this filter, are shown. An elevation of the ST segment may be seen, which is already evident after summing the first 10 harmonics (image at upper right).

 The introduction of high-pass filters with cut-off frequencies of 0.5 and 0.05 Hz yields the attenuations and phase shifts shown in Figure 2. In the frequency spectrum of our signal, a 0.5 Hz high-pass filter induces discrete amplitude attenuation of the first harmonic, though primarily and most importantly it causes phase shifts in up to the fifth or sixth harmonic (phase nonlinearities). As a consequence of the shifts produced in these first harmonics, summation of all of them would induce alterations in the ST segment that already become evident after the sum of the 10 first (Figure 10). On the other hand, 0.05 Hz high-pass filters cause neither attenuation nor phase shifting in our frequency spectrum, and so the shape of the resynthesized signal is unaffected.

## 4. Discussion

 The ECG may be regarded as a periodic signal whose cycle repeats starting from a P wave and extending to the following one. Consequently, according to Fourier's theorem, the signal can be expressed as the sum of infinite sine waves. Each of them exhibits certain amplitude and a frequency and constitutes an integral multiple of a fundamental frequency [4]. It may be shown that the value of this fundamental frequency equals the inverse of the period of repetition (*T*) of the original signal. Thus, the component with the smallest frequency has a frequency of 1/*T*, where *T* is the RR interval (see Figure 1). If the average value of an ECG signal is different from zero (this being the case in practice), it will also be necessary to sum a constant value known as the “continuous component,” which does not change the shape of the signal in any way and only produces a vertical displacement. Consideration of this continuous component was therefore not made in the present study.

 If *f*(*t*) represents the original ECG signal, it may be mathematically decomposed as

(1)f(t)=C0+∑n=0n=∞Cncos⁡(nwt+Φn),

where *C*_0_ is the continuous component, *C*_*n*_ the coefficient of the *n*th harmonic component, and Φ_*n*_ represents the corresponding phase. 

The amplitudes of the harmonic components (i.e., of the sine waves) decrease with *n*, as is seen in the frequency spectrum (Figure 1). Consequently, the smallest frequency components are those that contribute most to the strength of the signal. For this reason, no appreciable distortion of the shape of an ECG will be produced when considering a finite number of components. As may be seen in Figure 9, the sum of the first 30–50 harmonics reproduces the original ECG signal almost perfectly, because the contribution of the remaining harmonic components may be considered insignificant in relation to signal shape and power.

The need to use high-pass filters stems from a technical problem. At the tissue-electrode interface, direct current potentials (0 Hz) up to 200 mV level are generated, over which the electrical cardiac signal (generally 3-4 mV) is superimposed. In order to amplify the signal without saturating the electronic components, it is necessary to eliminate this continuous component. This effect can be achieved by using a high-pass filter. The use of 0.5 Hz high-pass filters attenuates the very low frequencies (0.1-0.2 Hz) that cause the baseline sway or wander and which are generally attributable to periodic respiratory movements (15 respirations/min yields a cycle of 4 seconds and a fundamental component of 0.25 Hz) but with the serious inconvenience of ST segment distortion.

For a heart rate of 40 bpm, the RR cycle (*T*) is 1.5 s and the first harmonic has a frequency of (1/1.5) 0.67 Hz. The remaining harmonics have frequencies that are integer multiples of this fundamental frequency (in this case, the second harmonic 0.67 × 2 = 1.34 Hz, the third harmonic 0.67 × 3 = 2.01 Hz, etc.). According to this and assuming that physiological heart rates are normally above 40 bpm, no biological components or signals attributable to an ECG will exist below 0.67 Hz.

All filtration causes attenuation (amplitude response, decrease of sinusoidal wave peak-to-peak amplitude) and/or phase shifts (phase response, phase shifting of the waves) that will affect one or other components according to the cut-off frequency of the filter used (Figure 2). Attenuations or phase shifts are produced from the cut-off frequency up to approximately 10 times this value. In our case, the attenuation caused by high-pass filtering with 0.5 Hz is minimal and only affects the first harmonic, as may be seen. However, it produces phase shifts in the first 5 to 6 harmonics (up to approximately 5 Hz), and these components are fundamental in the constitution of an ECG tracing (Figure 2). Logically, if phase shifts or displacements are produced in these first harmonics, their point-by-point sum (with all the others without phase shift) will change, and this alteration will distort the ST segment. In contrast, 0.05 Hz high-pass filters produce phase-shift harmonics up to approximately 0.5 Hz, a range where no intrinsic bioelectric signals exist, and, therefore, the shape of the ST segment remains unaffected (the first harmonic of our ECG was found at 0.9 Hz).

In 1966, Berson and Pipberger [5] studied alterations in the ST segment by analyzing the electrocardiographic signal with filters of 0.05, 0.1, 0.2, 0.3, and 0.5 Hz and in addition using three different attenuation slopes for each of these five cut-off frequencies. They pointed out that attenuation of low frequency harmonics were the cause of distortion and error in the interpretation of the ST segment. Moreover, such alterations were seen to be dependent on the cut-off frequency, and were more evident as this frequency increased. As in our study, modifications were more significant in ECGs with basal depolarization or repolarization alterations.

Based on these observations, in 1975 [10] the American Heart Association (AHA) developed technical specifications regarding electrocardiography and vectorcardiography in which they recommended a bandpass filter from 0.05 to 100 Hz for the purpose of ensuring adequate reproduction of ECG signals. Specifically, the filtered signal response should be flat (zero attenuation) between 0.14 and 50 Hz (level variations smaller than ±6% or ±0.5 dB), the attenuation at 0.05 and at 100 Hz being no greater than 30% (−3 dB). In this context, “dB” is a measure of the attenuation produced, and equals 20 × log_10_ [output amplitude/input amplitude]. It may easily be shown that if the input and output current amplitude is 100 and 70, respectively (corresponding to an attenuation of 30%), then 20 × log_10_ (70/100) = −3 dB. There was no specific reference made to phase linearity.

Subsequent research by Bragg-Remschel et al. [6] and Tayler and Vincent [7, 8] in the eighties demonstrated that distortions observed in the ST segment were basically caused by an inadequate phase response. In fact, even though attenuation was null, important distortions in the ST segment appeared. The authors proved that these distortions were due to nonlinear phase responses, that is, phase shifts in the low-frequency harmonics. The degree of distortion depended on the phase shift introduced and on the amplitude harmonics affected. This effect was more prominent in patients with lower heart rates (larger RR intervals, implying a lower-frequency fundamental component and a larger number of low-frequency harmonics), as well as that in cases with a wide QRS and slight basal deviation of the ST segment. In agreement with our own findings, leads exhibiting a dominant “S” wave showed elevations in the proximal ST portion, with terminal inversions of the T wave, while other leads with a dominant “R” wave exhibited ST segment depressions.

All these considerations, together with the development of biological signal digital processing technology, were reflected in the recommendations of the AHA [11] published in February 1990. These recommendations make special reference to the fact that high-pass filters with a cut-off frequency of 0.5 Hz may distort the ECG signal, affecting particularly the ST segment and T wave, while 0.05 Hz high-pass filters do not modify the resulting ECG shape. In conclusion, the AHA recommended a flat amplitude response (±6%, 0.5 dB) between 1 and 30 Hz, with an attenuation of −3 dB in frequencies ≤0.67 Hz and ≥150 Hz, also with some response characteristics for certain stimuli. The committee warned that low-pass filters (40, 100, 125, or 150 Hz) may give rise to small modifications in QRS amplitude, as well as distortions in rapid deflections or notches observed in QRS, although without altering either the ST segments or T waves. Figure 9 clearly shows that higher frequency components can affect notches, rapid components, and QRS amplitude but not ventricular repolarization.

It is essential that all the components passing through the recorder be delayed equally; that is, that phase response is linear over the physiological range of frequencies. High-pass filters with low cut-off frequencies exhibit phase distortion (phase nonlinearities) which describes the fact that a filter delays components of different frequencies by different amounts so that a distortion results when the signal components are summed together again [4, 8, 9].

The results and conclusions derived from the present study are interesting for two reasons. Firstly, because there is a lack of knowledge concerning the importance that the use of the filters may have in electrocardiography, this limitation is probably related to limited diffusion or understanding of the articles and guidelines referred to above. Indeed, none of the commonly used electrocardiographic textbooks make any reference to the importance and consequences of signal filtering. In the same way, Kligfield and Okin have recently described that the 75% of the routine ECGs did not meet the frequency bandwidth standard recommended by the AHA [12]. On the other hand, the ST segment distortion is visualized in manual mode (real-time record) but not seconds later in auto mode (computer processed) with the same filtering (i.e., 0.5–150 Hz). Digital technology allows correction of phase shifts through a linear phase filter. A common way to implement a linear phase filter is with a bidirectional filter. This filter applies the same high-pass filter in the forward direction, stores the data in memory, and passes the signal through the filter backward again, processing in a reverse-time direction, thus canceling phase distortion [4, 9]. In other words, the signal is processed differently (although in both, 0.5–150 Hz). A bidirectional filter cannot happen in real time. To prevent ST distortion, we can use a linear phase filter (computer processed) or a high-pass filter with a cut-off frequency of 0.05 Hz (in real-time recording).

Potentially serious diagnosis errors may be due to high-pass filters in real-time recording which may distort the ST segment and mimic acute coronary syndromes or Brugada's syndrome. This problem is especially relevant in external defibrillators used in ambulances, monitoring of ST segment in emergency department or chest pain units, telemetry, Holter recordings, or routine “real time” ECG with high-pass filter of 0.5 Hz.

All these considerations are reflected in the latest recommendations of the AHA [13], published in February 2007 that said “with traditional analogy filtering, a 0.5-Hz low-frequency cut-off introduces considerable distortion into the ECG, particularly with respect to the level of the ST segment. This distortion results from phase nonlinearities that occur in areas of the ECG signal where frequency content and wave amplitude change abruptly, as occurs where the end of the QRS complex meets the ST segment. Digital filtering provides methods for increasing the low-frequency cutoff without the introduction of phase distortion. This approach can be applied to ECG signals that are stored in computer memory, but it is not possible to achieve continuous real-time monitoring without a time lag.”

**Study Limitations**

As electrocardiograph manufacturers have different types of filters, the results will not apply to all available models.

## 5. Conclusions

 Technical characteristics of the recording mode influence the shape of the ST segment and may lead to confusion with acute coronary syndrome. Such variations are exclusively produced in the “real time” recording mode with high-pass filters of 0.5 Hz. Thus, the interpretation of the ST segment in this mode should only be carried out using high-pass filters of 0.05 Hz. It must be highlighted that, during the auto recording mode (computer processed with linear phase filter), the electrocardiograph compensates for any elevations or depressions of the ST segment by generating a processed and corrected signal, and thereby avoiding any effect upon the QRS repolarization.

## Figures and Tables

**Figure 7:**
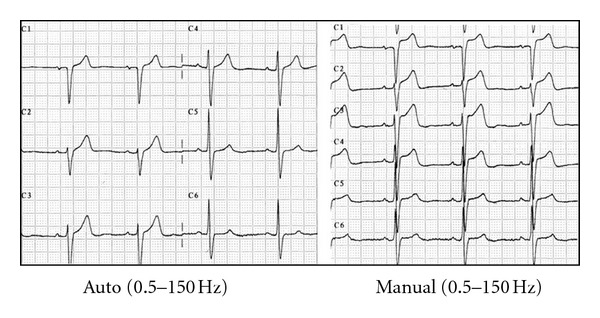
ECG selected for signal sampling. The shape of ventricular repolarization, superior concave in V1–V3, was likewise associated with significant variations of the ST in manual recording mode. Auto and manual mode (see Figure 3).

## Abbreviations

ST: ST segment
ECG: Electrocardiogram
Hz: Hertz
CLBB: Complete left bundle block
PP: P wave-P wave
RR: QRS complex-QRS complex
AHA: American Heart Association
dB: Decibel
mV: Millivolt
mm: Millimeter.
